# Research on quality changes of grass carp (*Ctenopharyngodon idellus*) during short‐term starvation

**DOI:** 10.1002/fsn3.1402

**Published:** 2020-01-19

**Authors:** Wenxian Yang, Wenzheng Shi, Yinghong Qu, Jiaying Qin, Zhihe Wang

**Affiliations:** ^1^ College of Food Science and Technology Shanghai Ocean University Shanghai China; ^2^ National R&D Branch Center for Freshwater Aquatic Products Processing Technology (Shanghai) Shanghai China

**Keywords:** adenosine triphosphate‐related compounds, electronic tongue, equivalent umami concentration, free amino acid, taste activity value

## Abstract

This study was aimed at to investigate the quality changes of grass carp during short‐term starvation. The pH, lactic acid, free amino acid, and adenosine triphosphate‐related compounds of dorsal meat, belly meat and red meat in grass carp were measured during starvation for 6 days, and the quality of grass carp was evaluated by *K* value, equivalent umami concentration (EUC), taste activity value (TAV), and electronic tongue. The pH of three parts meat reached the maximum value on the fourth day, which was closely related to the lactic acid content. Concurrently, the contents of fresh sweet amino acids were higher on the fourth day in all parts. The *K* values in dorsal meat and belly meat were below 10% during starvation. Considering the overall results of electronic tongue, EUC, and TAV analysis, it is suggested that grass carp should be marketed and eaten with a starvation period of 2–4 days for best taste and quality.

## Graphical abstract

This study was conducted to investigate the quality changes of grass carp meat, especially the taste quality as affected by short‐term starvation. The pH, lactic acid, adenosine triphosphate‐related compounds, *K* value, and levels of free amino acid were evaluated in grass carp meat with different starvation periods, so as to provide useful information for the quality improvement of grass carp.

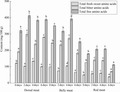

## 1 INTRODUCTION

Grass carp (*Ctenopharyngodon idellus*) is one of the main economic freshwater fish in China. Total global production of grass carp was about 6.06 million tons in 2016 (FAO, 2018), the highest for any aquaculture species. Because of its fast growth rate, easy cultivation, high nutritional value, high feed efficiency, and relatively low price, grass carp is favored by consumers. Aquatic products provide not only nutritious substances but also distinctive taste. With the development of economy and the change of lifestyle, the requirements of consumers for tasty, nutritious, and healthy aquatic products are also increasing. Therefore, more research on the quality changes as affected by different antemortem and postmortem treatments is necessary to satisfy the needs of consumers for aquatic products with high quality, which is of profound significance for the development of aquaculture and processing industry.

During the growth of grass carp, changes of protein, amino acid, and fat and other nutrients results in variations in flavor including taste and odor (Deng, Wang, & Liu, 2010). Taste‐active substances in fish are water soluble, among which free amino acids, nucleotides, small peptides, and minerals are the main components (Jiang & Su, 2008). Free amino acids play a very important role in maintaining the protein homeostasis of fish tissue and body, and are also essential for flavor indicators (Wang, Shi, Qiu, & Wang, 2018; Wang, Zhu, Zhang, Wang, & Shi, 2018). The components of free amino acids contribute a lot to taste, and they can interact with nucleotides to promote each other (Shirai et al., 1997). For example, the interaction between glutamate and creatinine in fish will produce substances with meat flavor characteristics. Nucleotides and their derivatives contribute greatly to the umami taste of fish (Yokoyama, Sakaguchi, Kawai, & Kanamori, 1992). After the death of fish, adenosine triphosphate (ATP) will degrade into adenosine diphosphate (ADP), adenosine monophosphate (AMP), and inosine monophosphate (IMP) with the catalysis by endogenous enzymes (Howgate, 2005). IMP is generally considered to be the main flavor component of fish and shellfish, and it will degrade to inosine (HxR) and hypoxanthine (Hx) which cause unpleasant flavor in fish (Tersaki, Kajikawa, & Fujita, 1965).

Freshwater fish is unacceptable to some people because of its special earthy smell. The earthy smell of freshwater fish is one of the main problems in the fishery industry. Numerous studies have demonstrated that temporary starvation with fresh water is effective to reduce the unpleasant odor in freshwater fish (Yang et al., 2009). According to the study of Zhou, Chen, and Yuan (2016), short‐term starvation can change the components and content of volatile substances in grass carp meat. It can also remove the bad flavor of tilapia meat and generate good odor (Du, Li, & Xiong, 2011). Studies have shown that the total volatile aldehydes of cod decreased significantly and the total volatile hydrocarbons increased during short‐term starvation (Palmeri, Turchini, Marriott, Morrison, & De Silva, 2009). In addition, short‐term starvation was reported to have a certain impact on the nutritional quality, edible quality, and texture characteristics of crucian carp (Wu, Chen, & Yuan, 2015), while it has no significant influence on muscle moisture, crude protein, and crude fat content of grass carp (Xia et al., 2017). Though the effects of short‐term starvation on the volatile components and physiological and biochemical properties of aquatic products have been studied in various fish species, including tilapia, carp, snapper, and bream (Abolfathi, Hajimoradloo, Ghorbani, & Zamani, 2012; Caruso et al., 2011; Fen et al., 2011; Liu et al., 2009), limited information is available on the effects of short‐term starvation on the quality of grass carp, especially the nonvolatile flavor substances. Therefore, this study was conducted to investigate the quality changes of grass carp meat, especially the taste quality as affected by short‐term starvation. The pH, lactic acid, ATP‐related compounds, *K* value, and levels of free amino acid were evaluated in grass carp meat with different starvation periods, so as to provide useful information for the quality improvement of grass carp.

## 2 MATERIALS AND METHODS

### 2.1 Sample preparation

Eighteen cultured grass carp (weight 2,500 ± 200 g) were captured from an aquaculture farm in Shanghai, China, in mid‐March in 2018, and transported to the laboratory alive within half an hour. Then, they were starved and four grass carp were randomly selected for experiment on starvation periods of 0, 2, 4, and 6 days. The carp were stunned by a blow to the head, scaled, gutted, filleted, and washed, and then, the fillets of dorsal meat, belly meat, and red meat were separated and packed in polyethylene bags after blending and stored at −80°C for further test.

### 2.2 pH

The determination of pH was using the following described method and be modified slightly (Wu et al., 2015). Within 45 min after fish slaughter, samples were weighed 2.00 g and added 18 ml of distilled water, respectively. The homogenate was centrifuged at 10,000 *g* for 10 min, and then, the supernatant was determined by pH meter. The average value of each sample was calculated by repeating the test three times.

### 2.3 Lactic acid

The determination of lactic acid was carried out according to the previously established method with minor modifications (Wang, Shi, et al., 2018; Wang, Zhu, et al., 2018). 2.00 g of samples were homogenized separately with 18 ml of cold 0.85% physiological saline for 60 s. The homogenate was centrifuged at 10,000 *g* for 10 min, and the supernatant was collected for further test. Protein concentration and the lactic acid content in the supernatant were determined by spectrophotometry at 530 and 595 nm, respectively, with assay kits (No. A019&A045, Nanjing Jiancheng Bioengineering Institute).

### 2.4 ATP‐related compounds and *K* value

The extraction of ATP‐related compounds was according to the described previously method, which was slightly modified (Yokoyama et al., 1992). All the operations were conducted below 4°C. The samples of grass carp meat were weighed 5.00 g and homogenized with 10 ml of cold perchloric acid, which volume fraction was 10%, by means of a FM‐200 homogenizer (Fluko Equipment Shanghai Co. Ltd) for 30 s, and centrifuged at 10,000 *g* for 15 min below 4°C. The precipitation was centrifuged under the same conditions after washing by 5 ml cold perchloric acid with a volume fraction of 5%, and then repeated this step twice and combined supernatant. The combined supernatant was adjusted pH to 6.5 with potassium hydroxide solution at 1 and 10 M concentrations, and then stilled for 30 min. The supernatant was diluted with high purity water to 10 ml and filtered with 0.45‐μm membrane. Then, the supernatant was detected and analyzed by high‐performance liquid chromatography (Shimadzu, LC‐10AT series) which was equipped with a COSMOSIL 5C18‐PAQ liquid chromatography column and a SPD‐10A (V) detector. Gradient elution was performed on samples of 10 μl using phosphoric acid buffer solution with a pH value of 6.5 and methanol at a flow rate of 1 ml/min. The detection wavelength was 254 nm. The *K* value was determined by the content of ATP, ADP, AMP, IMP, HxR, and Hx. The calculation formula is as follows: *K* value (%) = [(HxR + Hx)/(ATP + ADP + AMP + IMP + HxR + Hx)] × 100.

### 2.5 Free amino acids

Free amino acids (FAA) were determined according to the method established by Chen, Chen, and Shi (2017) with minor modifications. All the operations were conducted below 4°C. 2.00 g of samples were homogenized with 15 ml of trichloroacetic acid solution with a volume fraction of 15% for 2 min and then centrifuged at 10,000 *g* for 15 min after stilling for 2 hr. Five milliliter of supernatant after filtration were taken to adjust pH with sodium hydroxide solution at 3 M concentrations. When the pH value was adjusted to 2.0, set the capacity to 10 ml, shook evenly, and then filtered with 0.22‐μm membrane. The filtrate determined and analyzed by an automatic amino acid analyzer (L‐8800, Hitachi).

Free amino acid analysis conditions: column was 4.6 mm × 150 mm (7 μm), and column temperature was 50°C. The mobile phase were of sodium citrate and citric acid buffer mixture (pH 3.2, 3.3, 4.0, 4.9) and ninhydrin buffer (4% [w/w]), and the flow rate were 0.4 and 0.35 ml/min, respectively.

### 2.6 Taste‐active value and equivalent umami concentration

Taste activity value (TAV) was the ratio of the content of each flavor substance in the sample to its corresponding taste threshold, reflecting the contribution of taste‐active substances in fish to its taste (Scharbert & Hofmann, 2005). TAV >1 indicated that the taste‐active substance contributes significantly to the taste of fish, and the value was proportional to the contribution (Shi, Fang, & Wu, 2017).

Equivalent umami concentration (EUC) referred to the umami intensity produced by a single MSG with the same concentration as the umami intensity produced by the synergistic effect of umami amino acids and flavor nucleotide mixture. EUC can directly express the synergistic freshening effect between flavor nucleotides and umami amino acids (Chen & Zhang, 2007). The relation between them can be expressed by the following formula (Yamaguchi, Yoshikawa, Ikeda, & Ninomiya, 2010):

$$\text{EUC}=\sum a_{i}b_{i}+1,218\left(\sum a_{i}b_{i}\right)\times \left(\sum a_{j}b_{j}\right)$$

In the formula, EUC stands for equivalent umami concentration (gMSG/100 g), *a_i_* stands for the concentration of umami amino acids (Asp or Glu) (g/100 g), *b_i_* stands for the relative freshness coefficient of umami amino acids relative to MSG (Glu: 1; Asp: 0.077), *a_j_* stands for the concentration of flavor nucleotide (5′‐IMP, 5′‐GMP, 5′‐AMP, 5′‐XMP) (g/100 g), *b_j_* stands for the relative freshness coefficient of flavor nucleotide to IMP (5′‐IMP: 1; 5′‐GMP: 2.3; 5′‐AMP: 0.18; 5′‐XMP: 0.61), and 1,218 stands for the synergy constant.

### 2.7 Electronic tongue

Electronic tongue (Alpha M.O.S., ASTREE) was used for taste analysis of different parts meat of grass carp. The different parts meat of the grass carp were weighed 2.00 g accurately and homogenized with 25 ml of high purity water, and then centrifuged at 10,000 *g* for 10 min below 4°C after stilling for 15 min. The supernatant was filtered and diluted with high purity water to 100 ml and then used the electronic tongue to detect and analyze.

The electronic tongue sensor was conditioned and calibrated with hydrochloric acid at 0.01 M concentrations, and its function and stability were tested properly before sample analysis. After successful calibration, 5 ml of prepared samples were taken for 16‐fold dilution, and then, each sample was analyzed seven times for 120 s each time. The data of the last three times were taken as the original data of principal component analysis (PCA). The obtained original data have the property of multivariable, which is expressed as the relationship between voltage and time (Raithore et al., 2015). To prevent carryover effects, the sensors were washed with deionized water after each measurement.

### 2.8 Statistical analysis

SPSS 25.0 (SPSS Inc.) software was used to analyze the variance of the data expressed as mean values ± standard deviations. The method of Duncan was used to represent the results of multiple comparisons. The software of Origin 8.5 (OriginLab Corp) was applied to draw. The electronic tongue data used the method of principal component analysis to obtain PCA diagram.

## 3 RESULTS AND DISCUSSION

### 3.1 Changes in pH

**Figure 1:**
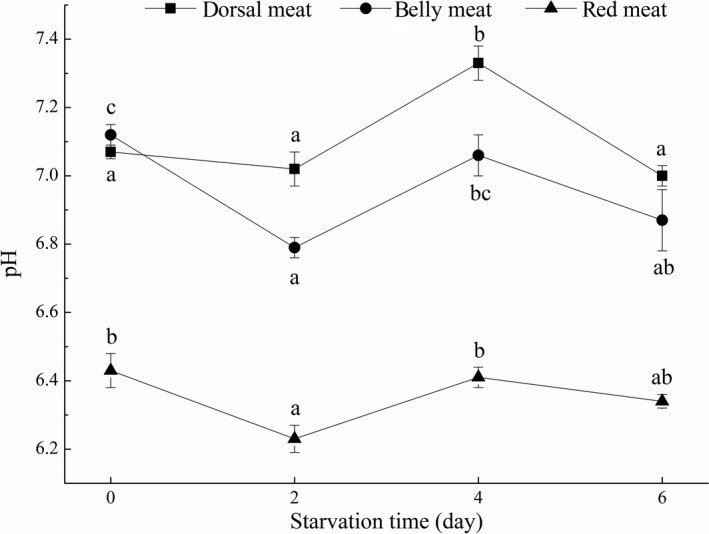
The pH of dorsal meat, belly meat, and red meat in grass carp during starvation for 0, 2, 4, and 6 days. Data (mean ± *SD*) with different letters in the same part meat are significantly different (*p* < .05)

The initial pH values of dorsal meat, belly meat, and red meat of grass carp were 7.07 ± 0.02, 7.12 ± 0.03, and 6.43 ± 0.05, respectively, and the pH values of red meat were lower than those of dorsal meat and belly meat during the whole process of short‐term starvation (Figure 1). Fang, Shi, Diao, Wang, and Wang (2018) showed that the pH value of red meat of grass carp was generally lower than that of dorsal meat and belly meat. This difference could be due to different metabolic pathways of glycogen in dorsal meat, belly meat, and red meat (Zhu & Shen, 2002).

During the whole process of short‐term starvation of grass carp, the pH of dorsal meat, belly meat, and red meat all decreased to lower values on the second day (7.02 ± 0.05, 6.79 ± 0.03, and 6.23 ± 0.04, respectively), then increased on the fourth day (7.33 ± 0.05, 7.06 ± 0.06, and 6.41 ± 0.03, respectively), and then decreased on the sixth day (7.00 ± 0.03, 6.87 ± 0.09, and 6.34 ± 0.02, respectively). The initial decrease in pH values may be due to the decomposition of glycogen in different parts. In addition, acidic substances produced by the reaction of substances such as adenosine triphosphate and inosine monophosphate also caused the decrease in pH value (Xiong, Peng, & Jin, 2012). The decrease in pH value would have an impact on the physical properties of muscle such as water retention, preservation, and processing characteristics. Therefore, pH could well reflect the quality of muscle (Fang et al., 2018). Subsequently, the rise in pH values may be due to the degradation of protein, which produced peptides, small peptides, and decarboxylate to produce amines, amino groups to produce NH3, and fats were oxidized to produce aldehydes, ketones, alkanes, and other substances (Delbarre‐Ladrat, Chéret, Taylor, & Verrez‐Bagnis, 2006). Some studies have shown that the increase in pH value will improve the quality of meat within a certain range (Xia et al., 2017).

### 3.2 Changes in lactic acid content

Lactic acid is mainly formed from glycogen via glycolysis when fish muscles are short of oxygen supply (Einen & Thomassen, 1998). Low dissolved oxygen content of water, slow blood circulation and different degrees of stress response in the process of fishing and transportation can lead to the increase of lactic acid content in the body (Jiang, Lin, & Wu, 2016). An increase in lactic acid could make the pH value decrease, leading to degeneration of muscle fibrin, and then leading to deterioration of fish quality (Lv, Chen, & Deng, 2001).

**Figure 2:**
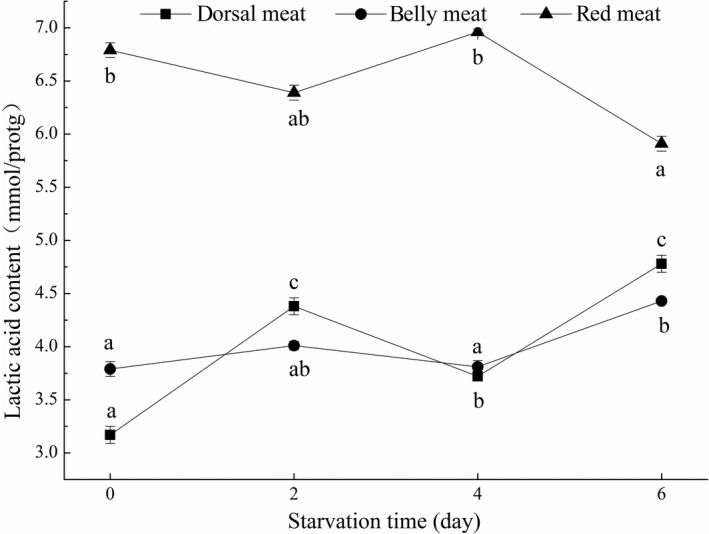
The lactic acid contents of dorsal meat, belly meat, and red meat in grass carp during starvation for 0, 2, 4, and 6 days. Data (mean ± *SD*) with different letters in the same part meat are significantly different (*p* < .05)

During the whole process of short‐term starvation of grass carp, the lactic acid content of red meat was significantly higher than that of dorsal meat and belly meat (Figure 2), and the contents were at 5.91–6.96 mmol/gprot, which was related to the change of glycogen content. The results were consistent with the research on the influence of different freezing methods on lactic acid content in different parts meat of grass carp (Fang et al., 2018) and the change of lactic acid content in grass carp of different sizes (Chen et al., 2017). The lactic acid content in red meat showed a trend of decrease on the sixth day, which may be due to the reason that grass carp needed a large amount of energy to maintain the metabolism of the body when they were starved, and they would give priority to the use of glycogen in red meat as energy material, and then, the glycolysis was enhanced (Shin & Sho, 1991). The lactic acid contents in dorsal meat and belly meat was close to 3.17–4.78 mmol/gprot and significantly decreased to 3.72 ± 0.04 mmol/gprot on the fourth day in dorsal meat. As a result, red meat was more prone to deterioration than dorsal meat and belly meat. There was a significant difference in lactic acid content during short‐term starvation (*p* < .05), which was negatively correlated with glycogen content and pH value. Wang, Shi, et al. (2018) studied that the trend of lactic acid content was increased first and then decreased during the postmortem at room temperature (25°C) of grass carp.

### 3.3 ATP‐related compounds and *K* value analysis

Adenosine triphosphate and its related compounds are widely used to detect freshness and shelf life of fish, and are also important flavor components in fish (Liu, Chen, Zhang, & Lu, 2014). After the death of the fish, ATP is degraded into ADP, AMP, IMP, HxR, and Hx successively due to the enzymatic catalysis of adenosine triphosphate (Howgate, 2005). From the Table 1, it was not difficult to see that there were differences in the content of nucleotide compounds in different parts meat of grass carp. The total amount of nucleotide compounds in dorsal meat and belly meat of grass carp were much higher than that in red meat, which was consistent with the results of relevant studies on the effect of freezing methods on the taste of grass carp in different parts meat (Fang et al., 2018). As can be seen from Table 1, the content of IMP in various parts was significantly higher than that of other related substances, which was because the AMP of fish was converted into HxR at a very slow rate during the degradation process, so IMP can be accumulated in a large amount in fresh fish (Hayashi, Yamaguchi, & Konosu, 1981). In addition, the IMP content of dorsal meat, belly meat, and red meat reached the maximum on the second day, which was 241.23 mg/100 g, 217.30 mg/100 g, and 115.08 mg/100 g, respectively. However, HxR content in dorsal meat and red meat decreased significantly on the second day and reached the minimum value of 4.42 mg/100 g and 14.00 mg/100 g, respectively. As is known to all, IMP is the main umami nucleotide in fish and the freshness of fresh grass carp meat is closely related to the high content of IMP (Takashi, Maya, Hideyuki, & Toshihiro, 2007). Hx is the final product of ATP degradation pathway, which was believed to have putrefying bitterness and negatively affected the flavor quality of grass carp (Tersaki et al., 1965).

**Table 1:** Changes of the contents of nucleotide compounds of dorsal meat, belly meat, and red meat in grass carp during short‐term starvation

Different parts	Starvation time (day)	Contents/(mg/100 g)						
		IMP	ATP	ADP	AMP	Hx	HxR	Total
Dorsal meat	0	244.73 ± 9.74^b^	1.29 ± 0.91^a^	16.80 ± 6.30^a^	1.21 ± 0.54^a^	0.01 ± 0.02^a^	10.95 ± 2.53^b^	274.99 ± 4.70^b^
	2	241.23 ± 8.02^b^	6.17 ± 6.47^a^	8.79 ± 5.53^a^	5.24 ± 2.56^b^	0.04 ± 0.06^a^	4.42 ± 0.64^a^	265.88 ± 22.79^b^
	4	210.60 ± 8.38^a^	6.38 ± 3.93^a^	8.77 ± 5.09^a^	5.52 ± 2.69^b^	0.38 ± 0.34^a^	6.02 ± 1.77^a^	237.66 ± 12.53^a^
	6	235.32 ± 6.69^b^	1.94 ± 1.55^a^	7.24 ± 2.76^a^	4.22 ± 0.57^ab^	0.43 ± 0.25^a^	4.17 ± 0.62^a^	253.33 ± 7.01^ab^
Belly meat	0	211.74 ± 13.86^b^	1.12 ± 0.57^a^	4.94 ± 1.45^a^	2.91 ± 0.59^a^	2.10 ± 1.26^a^	10.20 ± 1.30^b^	233.01 ± 13.12^ab^
	2	217.30 ± 16.45^b^	5.16 ± 2.00^b^	6.80 ± 3.41^a^	5.09 ± 2.82^a^	6.99 ± 4.18^a^	9.43 ± 0.41^ab^	250.76 ± 17.86^b^
	4	189.39 ± 15.69^ab^	4.06 ± 1.37^ab^	3.97 ± 0.97^a^	4.93 ± 2.50^a^	2.16 ± 0.74^a^	7.70 ± 0.36^ab^	212.20 ± 18.83^a^
	6	175.08 ± 21.36^a^	3.30 ± 1.96^ab^	5.98 ± 3.22^a^	3.09 ± 2.76^a^	5.84 ± 5.52^a^	6.68 ± 2.88^a^	199.97 ± 17.10^a^
Red meat	0	98.29 ± 15.78^bc^	5.88 ± 3.17^a^	6.02 ± 4.67^a^	5.49 ± 5.97^a^	4.99 ± 1.67^ab^	18.20 ± 2.70^b^	138.87 ± 30.29^a^
	2	115.08 ± 30.09^c^	3.82 ± 3.10^a^	8.16 ± 3.27^a^	6.56 ± 2.59^a^	4.07 ± 1.09^a^	14.00 ± 2.55^a^	151.69 ± 38.49^a^
	4	78.88 ± 7.66^ab^	5.63 ± 4.47^a^	4.85 ± 3.99^a^	6.23 ± 2.95^a^	5.97 ± 0.44^ab^	21.60 ± 1.93^b^	123.16 ± 17.74^a^
	6	63.98 ± 0.50^a^	0.70 ± 0.48^a^	3.56 ± 0.55^a^	5.49 ± 1.32^a^	7.25 ± 1.13^b^	22.40 ± 1.14^b^	103.37 ± 1.29^a^

Note

Different superscript letters in the same column and same part indicate a significant difference (*p* < .05) between different time.

**Figure 3:**
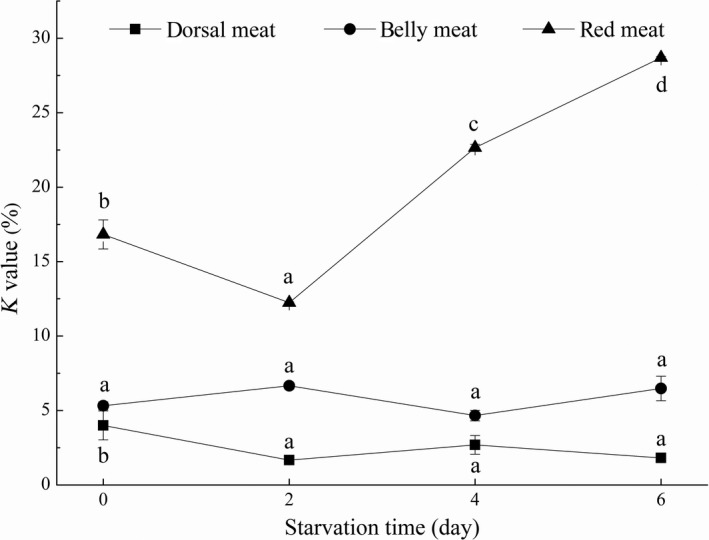
The *K* value of dorsal meat, belly meat, and red meat in grass carp during starvation for 0, 2, 4, and 6 days. Data (mean ± *SD*) with different letters in the same part meat are significantly different (*p* < .05)

*K* value is a biochemical indicator reflecting freshness and flavor of fish. The smaller *K* value is, the better the freshness is (Aubourg et al., 2007). During the whole short‐term starvation process of grass carp, the *K* values of dorsal meat and belly meat were <10% (Figure 3), which was very fresh and consistent with the results of different parts meat of grass carp of different sizes studied (Chen et al., 2017). The *K* value of dorsal meat and red meat decreased significantly on the second day ([1.67 ± 0.12]% and [12.24 ± 0.04]%, respectively), and there was no significant change in belly meat, indicating that short‐term starvation could improve the freshness of grass carp to some extent. However, the *K* value of red meat significantly increased on the fourth day, which may be caused by the different energy metabolism of red meat and other two parts meat under the case of starvation. Studies have also shown that *K* value is not suitable for evaluating the freshness of red meat (Chen et al., 2017).

**Figure 4:**
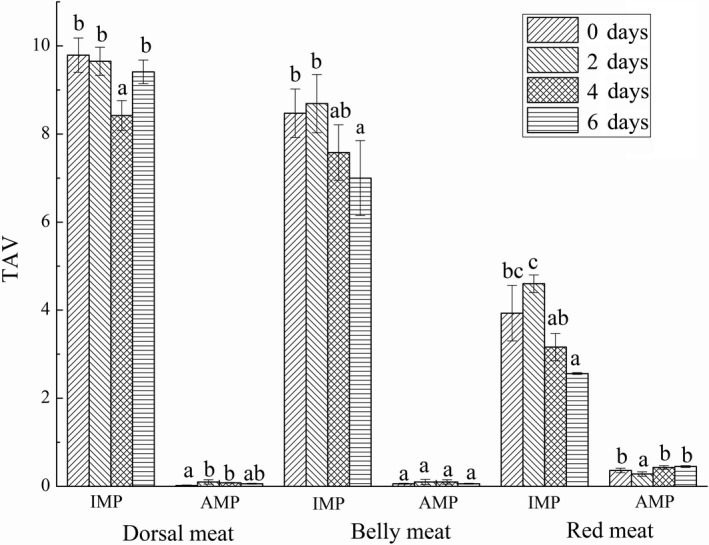
Taste activity value (TAV) of the nucleotides of dorsal meat, belly meat, and red meat in grass carp during starvation for 0, 2, 4, and 6 days. Data (mean ± *SD*) with different letters in the same part meat are significantly different (*p* < .05)

Inosine monophosphate was the highest nucleotide content in grass carp (Table 1), and the content was much higher than its threshold (25 mg/100 g). As can be clearly seen from Figure 4, TAV of IMP was >1 in all samples, while TAV of AMP was <1, indicating that IMP had a significant impact on the overall taste of grass carp and contributed more to the taste than AMP. The flavor contributions were different from the shrimp (Li & Chen, 2015) and crab (Chen & Zhang, 2007), showing that the flavor nucleotide content was related to the types of aquatic products.

### 3.4 FAA analysis

Free amino acids are important flavor components in aquatic products, and the flavoring characteristics are related to their content, threshold, and interaction with other flavoring components (Deng et al., 2010). When certain free amino acids reach a high enough concentration in fish, they can act independently of other components on the overall flavor of fish. Studies have shown that Asp, Glu, and Gly have umami taste, while Lys and His have bitter taste (Ren & Zhang, 2003).

The changes of free amino acid content in grass carp meat during short‐term starvation were listed in Tables 2, 3, 4. It was found that the free amino acids with high content in each part meat of grass carp during short‐term starvation included Thr, Gly, Ala, Lys, and His, which was consistent with existing research results on grass carp (Fang et al., 2018). It was not difficult to see that the contents of Thr, Gly, Ala, and Lys in various parts of meat of grass carp increased to higher level on the fourth day, which in dorsal meat was 19.06 mg/100 g, 80.17 mg/100 g, 35.29 mg/100 g, and 22.72 mg/100 g, respectively (Table 2). Their contents in belly meat were 21.50 mg/100 g, 58.01 mg/100 g, 29.34 mg/100 g, and 25.88 mg/100 g (Table 3) and, in red meat, were 15.45 mg/100 g, 14.19 mg/100 g, 21.48 mg/100 g, and 21.53 mg/100 g, respectively (Table 4), on the fourth day. Further analysis showed that the contents of Thr, Glu, and Ala in dorsal meat and red meat of grass carp had the largest increase rate on the second day, with 144.46%, 67.35%, and 254.96% in dorsal meat, 62.13%, 51.09%, and 44.16% in red meat, respectively. This indicated that grass carp may taste better during the starvation period of 2–4 days. The content of His in grass carp meat was higher than its threshold value of 20 mg/100 g, and the contents of dorsal meat and belly meat were much higher than that of red meat, so this may be the reason for the different flavor of dorsal meat, belly meat, and red meat.

**Table 2:** Changes of free amino acid contents of dorsal meat in grass carp during short‐term starvation

Amino acid species	Taste characteristics	Threshold (mg/100 g)	Content/(mg/100 g)			
			0 day	2 days	4 days	6 days
Asp†	Fresh/sweet (+)	3	0.36 ± 0.06^a^	0.59 ± 0.01^b^	0.63 ± 0.08^b^	0.84 ± 0.03^c^
Thr‡	Sweet (+)	260	9.02 ± 0.06^a^	22.05 ± 0.65^b^	19.06 ± 2.86^b^	21.73 ± 0.31^b^
Ser†	Sweet (+)	150	3.65 ± 0.09^a^	7.87 ± 0.24^b^	9.83 ± 0.11^c^	10.61 ± 0.05^d^
Glu†	Fresh (+)	30	0.98 ± 0.36^a^	1.64 ± 0.07^bc^	1.40 ± 0.08^ab^	2.10 ± 0.11^c^
Gly†	Sweet (+)	130	85.28 ± 0.35^d^	61.97 ± 0.82^b^	80.17 ± 1.07^c^	52.48 ± 2.04^a^
Ala†	Sweet (+)	60	9.35 ± 1.85^a^	24.67 ± 0.75^b^	35.29 ± 0.55^d^	28.23 ± 0.77^c^
Val‡	Sweet/bitter (−)	40	6.95 ± 1.51^a^	12.62 ± 0.38^b^	14.05 ± 0.17^b^	17.91 ± 0.03^c^
Met	Bitter/sweet/sulfur (−)	30	3.00 ± 1.77^a^	3.54 ± 0.17^a^	5.04 ± 0.02^a^	3.86 ± 0.41^a^
Ile‡	Bitter (−)	90	4.44 ± 2.28^a^	7.61 ± 0.20^ab^	9.54 ± 0.05^b^	9.68 ± 0.40^b^
Leu‡	Bitter (−)	190	5.66 ± 1.38^a^	11.86 ± 0.28^b^	14.89 ± 0.17^c^	14.92 ± 0.38^c^
Tyr	Bitter (−)	ND	4.36 ± 1.86^a^	7.00 ± 0.03^b^	7.30 ± 0.08^b^	4.32 ± 0.20^a^
Phe‡	Bitter (−)	90	2.90 ± 0.99^a^	4.54 ± 0.35^b^	6.18 ± 0.02^c^	4.63 ± 0.22^b^
Lys‡	Sweet/bitter (−)	50	12.40 ± 0.79^a^	23.82 ± 0.68^b^	22.72 ± 0.52^b^	50.34 ± 0.29^c^
His‡	Bitter (−)	20	145.62 ± 1.37^a^	206.10 ± 8.42^b^	138.67 ± 1.80^a^	144.63 ± 0.72^a^
Arg‡	Sweet/bitter (+)	50	5.46 ± 1.28^a^	10.43 ± 0.43^b^	13.42 ± 0.30^c^	16.74 ± 0.29^d^
Pro†	Sweet/bitter (+)	300	22.04 ± 1.18^b^	6.69 ± 0.46^a^	6.60 ± 0.13^a^	6.19 ± 0.03^a^
TFAA			321.46 ± 12.71^a^	413.01 ± 13.86^b^	384.78 ± 7.47^b^	389.21 ± 6.22^b^

Note

ND indicates that the threshold was not detected; Different superscript letters in the same row show that the index was significant different (*p* < .05) at different time.

^†^ Fresh, sweet amino acids.

^‡^ Bitter amino acids.

**Table 3:** Changes of free amino acid contents in belly meat of grass carp during short‐term starvation

Amino acid species	Taste characteristics	Threshold (mg/100 g)	Content/(mg/100 g)			
			0 day	2 days	4 days	6 days
Asp†	Fresh/sweet (+)	3	0.50 ± 0.02^a^	0.60 ± 0.00^b^	0.67 ± 0.02^c^	0.63 ± 0.04^bc^
Thr‡	Sweet (+)	260	16.99 ± 0.07^b^	16.94 ± 0.03^b^	21.50 ± 0.57^c^	14.57 ± 0.13^a^
Ser†	Sweet (+)	150	5.07 ± 0.09^a^	6.02 ± 0.02^b^	9.20 ± 0.19^d^	7.46 ± 0.03^c^
Glu†	Fresh (+)	30	1.24 ± 0.21^a^	1.29 ± 0.11^a^	1.46 ± 0.06^ab^	1.89 ± 0.22^b^
Gly†	Sweet (+)	130	54.75 ± 0.37^c^	41.61 ± 0.01^b^	58.01 ± 1.54^d^	32.90 ± 0.26^a^
Ala†	Sweet (+)	60	23.14 ± 0.10^c^	20.27 ± 0.56^b^	29.34 ± 0.83^d^	18.11 ± 0.19^a^
Val‡	Sweet/bitter (−)	40	9.29 ± 0.05^a^	11.15 ± 0.71^b^	15.04 ± 0.53^c^	14.44 ± 0.43^c^
Met	Bitter/sweet/sulfur (−)	30	2.25 ± 0.10^a^	3.09 ± 0.35^b^	4.62 ± 0.24^c^	2.43 ± 0.36^ab^
Ile‡	Bitter (−)	90	4.41 ± 0.22^a^	6.37 ± 0.41^b^	8.75 ± 0.30^c^	6.77 ± 0.61^b^
Leu‡	Bitter (−)	190	6.33 ± 0.10^a^	9.71 ± 0.24^b^	14.12 ± 0.39^c^	10.49 ± 0.55^b^
Tyr	Bitter (−)	ND	4.35 ± 0.54^b^	5.79 ± 0.27^c^	9.45 ± 0.31^d^	3.08 ± 0.16^a^
Phe‡	Bitter (−)	90	3.05 ± 0.04^a^	3.67 ± 0.22^b^	5.34 ± 0.03^c^	3.47 ± 0.28^ab^
Lys‡	Sweet/bitter (−)	50	16.50 ± 1.40^a^	18.07 ± 0.26^a^	25.88 ± 1.04^b^	31.06 ± 0.37^c^
His‡	Bitter (−)	20	180.07 ± 0.03^c^	161.07 ± 5.06^b^	169.92 ± 4.68^bc^	91.46 ± 4.93^a^
Arg‡	Sweet/bitter (+)	50	8.40 ± 0.24^a^	7.65 ± 0.20^a^	12.98 ± 0.57^c^	10.61 ± 0.07^b^
Pro†	Sweet/bitter (+)	300	14.71 ± 0.01^d^	5.03 ± 0.10^b^	6.76 ± 0.15^c^	4.24 ± 0.08^a^
TFAA			351.07 ± 0.58^c^	318.31 ± 8.26^b^	393.03 ± 11.38^d^	253.59 ± 7.57^a^

Note

ND indicates that the threshold was not detected; Different superscript letters in the same row show that the index was significant different (*p* < .05) at different time.

^†^ Fresh, sweet amino acids.

^‡^ Bitter amino acids.

**Table 4:** Changes of free amino acid contents in red meat of grass carp during short‐term starvation

Amino acid species	Taste characteristics	Threshold (mg/100 g)	Content/(mg/100 g)			
			0 day	2 days	4 days	6 days
Asp†	Fresh/sweet (+)	3	2.83 ± 0.03^b^	2.69 ± 0.20^ab^	4.78 ± 0.16^c^	2.39 ± 0.07^a^
Thr‡	Sweet (+)	260	8.82 ± 0.21^a^	14.30 ± 0.36^b^	15.45 ± 0.47^c^	9.59 ± 0.31^a^
Ser†	Sweet (+)	150	3.78 ± 0.08^a^	4.92 ± 0.19^b^	5.82 ± 0.05^c^	4.19 ± 0.34^a^
Glu†	Fresh (+)	30	6.42 ± 1.11^a^	9.70 ± 1.35^b^	10.37 ± 0.40^b^	6.88 ± 0.24^a^
Gly†	Sweet (+)	130	15.50 ± 0.02^c^	17.37 ± 0.27^d^	14.19 ± 0.15^b^	9.22 ± 0.62^a^
Ala†	Sweet (+)	60	12.75 ± 0.39^a^	18.38 ± 0.00^b^	21.48 ± 0.65^c^	13.77 ± 0.12^a^
Val‡	Sweet/bitter (−)	40	4.19 ± 0.14^a^	7.62 ± 0.44^d^	6.74 ± 0.14^c^	5.43 ± 0.01^b^
Met	Bitter/sweet/sulfur (−)	30	1.28 ± 0.01^a^	2.51 ± 1.11^a^	2.11 ± 0.02^a^	1.42 ± 0.08^a^
Ile‡	Bitter (−)	90	2.64 ± 0.23^a^	7.20 ± 1.58^b^	5.40 ± 0.19^b^	5.00 ± 0.35^b^
Leu‡	Bitter (−)	190	4.38 ± 0.21^a^	10.11 ± 1.24^c^	8.99 ± 0.24^bc^	7.72 ± 0.11^b^
Tyr	Bitter (−)	ND	3.07 ± 0.07^a^	7.31 ± 2.83^b^	8.51 ± 0.23^b^	2.50 ± 0.28^a^
Phe‡	Bitter (−)	90	2.21 ± 0.25^a^	4.65 ± 1.70^a^	3.31 ± 0.05^a^	2.98 ± 0.23^a^
Lys‡	Sweet/bitter (−)	50	13.09 ± 0.02^b^	15.80 ± 0.23^c^	21.53 ± 0.82^d^	7.75 ± 0.79^a^
His‡	Bitter (−)	20	85.68 ± 0.05^d^	75.51 ± 0.95^c^	68.57 ± 0.90^b^	27.72 ± 2.16^a^
Arg‡	Sweet/bitter (+)	50	4.15 ± 0.11^b^	5.42 ± 0.06^c^	7.90 ± 0.29^d^	3.43 ± 0.26^a^
Pro†	Sweet/bitter (+)	300	8.54 ± 0.03^c^	4.00 ± 0.13^b^	3.96 ± 0.09^b^	2.02 ± 0.19^a^
TFAA			179.33 ± 1.40^b^	207.49 ± 8.05^c^	209.10 ± 4.55^c^	112.00 ± 3.27^a^

Note

ND indicates that the threshold was not detected; Different superscript letters in the same row show that the index was significant different (*p* < .05) at different time.

^†^ Fresh, sweet amino acids.

^‡^ Bitter amino acids.

**Figure 5:**
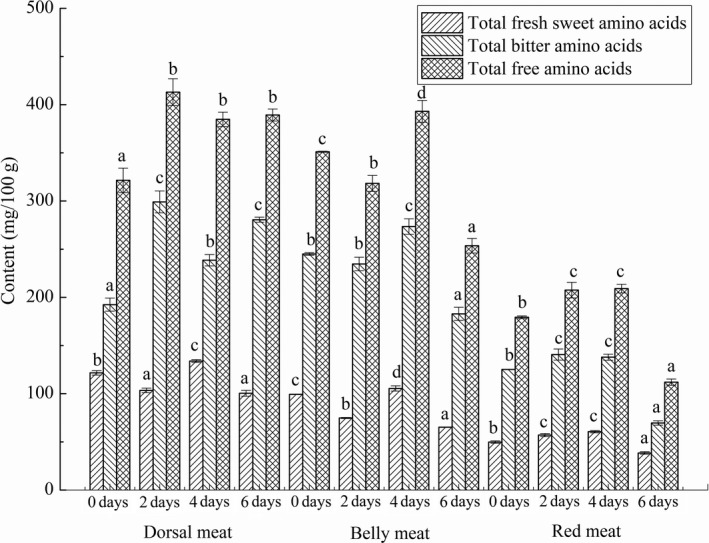
The free amino acids contents of dorsal meat, belly meat, and red meat in grass carp during starvation for 0, 2, 4, and 6 days. Data (mean ± *SD*) with different letters in the same part meat are significantly different (*p* < .05)

Figure 5 showed the contents of total fresh sweet amino acids, total bitter amino acids, and total free amino acids during short‐term starvation. The content of total free amino acids in dorsal meat reached a maximum of 413.01 ± 13.86 mg/100 g on the second day and that in belly meat and red meat reached a maximum on the fourth day, at 393.03 ± 11.38 mg/100 g and 209.10 ± 4.55 mg/100 g, respectively. The content of bitter amino acid was opposite to that of fresh sweet amino acid. As can be seen from Figure 5, the contents of fresh sweet amino acids in red meat of grass carp were significantly lower than that in dorsal meat and belly meat. Chen, Shi, and Shao (2016) studied the effects of different slaughter methods on the content of flavor amino acids and found that the content of bitter amino acids in the blood of fish accounted for up to 70% of the free amino acids. Therefore, during the slaughter of grass carp, the infiltration of fish blood into the muscle would affect the proportion of fresh and sweet amino acids and bitter amino acids in the muscle. These differences in free amino acids may also be related to temperature (Hwang, Chen, Shiau, & Jeng, 2000).

### 3.5 EUC and TAV analysis

**Figure 6:**
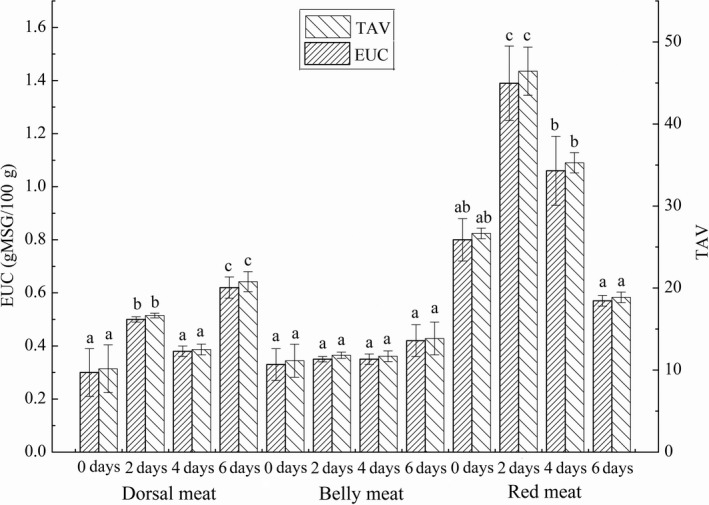
The EUC value and TAV of dorsal meat, belly meat, and red meat in grass carp during starvation for 0, 2, 4, and 6 days. Data (mean ± *SD*) with different letters in the same part meat are significantly different (*p* < .05)

Equivalent umami concentration was usually used to evaluate the synergistic freshening effect of umami amino acids and flavoring nucleotides. The synergistic effect of AMP and sodium glutamate at a certain concentration could improve the umami of aquatic products, and the synergistic effect with IMP could also improve the overall umami of aquatic products (Shirai et al., 1997). The umami amino acids in grass carp meat were Asp and Glu, and the flavoring nucleotides were IMP and AMP. Figure 6 showed the changes of EUC value and TAV of each part meat in grass carp during different starvation time. It was found that EUC value and TAV of red meat in grass carp were higher than those of other two parts meat, and EUC value reached a maximum of 1.39 ± 0.14 gMSG/100 g on the second day, indicating that starvation for 2 days could improve the umami of red meat in grass carp. The EUC value of belly meat did not change significantly during the period of short‐term starvation. The EUC value of dorsal meat increased on the second day, but was far less than that of red meat, indicating that red meat had a greater contribution to the overall umami of grass carp. The EUC value of the same aquatic product may vary with different parts. Study have been found that the EUC value of the hepatopancreas and gonads of Eriocheir sinensis was 2.73 gMSG/100 g and 56.07 gMSG/100 g, respectively (Peng, Zhou, & Wang, 2019).

### 3.6 Electronic tongue analysis

**Figure 7:**
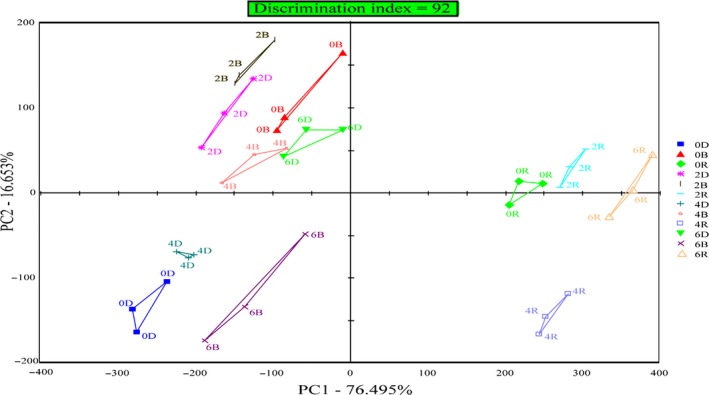
PCA analysis of E‐tongue results recorded during short‐term starvation. 0D, 0B, 0R, 2D, 2B, 2R, 4D, 4B, 4R, 6D, 6B, and 6R represent 0, 2, 4, and 6 days of starvation, respectively, for dorsal, belly, and red meat

Principal component analysis (PCA) was used to analyze the taste of different parts of grass carp (Figure 7). The result of principal component analysis was a two‐dimensional scatter diagram composed of PC1 and PC2 axes. The contribution rate of the principal component in the PCA plot represented the original amount of information contained in the principal component. The larger the contribution rate, that was, the total contribution rate exceeded 85% (Raithore et al., 2015), the more fully the information was reflected the original multi‐index information by the principal component. As can be seen from Figure 7, the contribution rates of the first principal component (PC1) and the second principal component (PC2) were, respectively, 76.50% and 16.65%, and the total contribution rate was 93.15%. The results showed that PCA could more accurately reflect the flavor change of grass carp during short‐term starvation.

As shown in Figure 7, the more dispersed the distribution of points in the figure, the greater the differences between samples. The difference of the area surrounded by the points of every three repeated samples represented the difference of the samples, and the size of the area reflected the ability of the electronic tongue to distinguish and recognize the sample (Legin, Rudnitskaya, Vlasov, Natale, & D'Amico, 1999). The nonoverlapping area in the PCA plot (Beullens et al., 2006) showed that the electronic tongue could clearly identify the differences between different parts meat of grass carp. At the same time, the DI of principal component obtained by the analysis software was 92, indicating that the taste of grass carp meat in different parts meat had obvious difference in different starvation time. The further analysis showed that the distribution of red meat during starvation was quite different from that of other two parts meat, which indicated that the taste of red meat was different from that of dorsal meat and belly meat in grass carp.

## 4 CONCLUSIONS

This study focused on the quality changes of grass carp during short‐term starvation. During the starvation period of 6 days, it was found that the pH change of each part meat was closely related to the lactic acid content of grass carp. The *K* value of dorsal meat and red meat decreased significantly on the second day, and the freshness was improved. In terms of the total amount of fresh sweet amino acids, combined with EUC and TAV, grass carp tasted best when they were starved for 2–4 days. The results showed that PCA could effectively highlight the taste changes of different parts meat of grass carp during different starvation periods. Overall, the quality of grass carp was best when they were starved for 2–4 days. These results provided useful information for improving the quality control of grass carp in aquaculture and production practice, and would help consumers to choose grass carp reasonably. In addition, the research on the enzymatic activity of grass carp during starvation can help us to understand and explore the taste and quality of grass carp, and thus help to develop more effective methods to improve the quality of grass carp.

## CONFLICT OF INTEREST

The authors declare that they do not have any conflict of interest.

## ETHICAL APPROVAL

This study does not involve any human or animal testing.
